# Determinants of Age at First Exposure to Digital Devices Among Children Aged 2 to 6 Years: Survival Analysis

**DOI:** 10.2196/100072

**Published:** 2026-10-28

**Authors:** Daniela Rodrigues, Gabriela Costa, Rita Gonçalves, Aristides M Machado-Rodrigues, Josep A Tur, Niko Männikkö

**Affiliations:** 1 Research Centre for Anthropology and Health, University of Coimbra, Ed São Bento, Calçada Martim de Freitas, Coimbra, Coimbra, 3000-456, Portugal, 351 239 240 700; 2 Faculty of Sport Sciences and Physical Education, University of Coimbra, Coimbra, Coimbra, Portugal; 3 Child and Adolescent Psychiatry Service, Pediatric Hospital, Hospitais da Universidade de Coimbra, Coimbra, Coimbra, Portugal; 4 Research Group on Community Nutrition & Oxidative Stress, Universitat de les Illes Balears-IUNICS, IDISBA & CIBEROBN, Palma de Mallorca, Balearic Islands, Spain; 5 Centre for Research and Innovation, Oulu University of Applied Sciences, Oulu, North Ostrobothnia, Finland

**Keywords:** children, screen use, first exposure, parental behavior, survival analysis

## Abstract

**Background:**

Digital media exposure is nearly universal by the preschool years. However, little is known about the timing of first exposure to different devices, and the factors associated with earlier exposure.

**Objective:**

This study examined age at first exposure to multiple digital devices and investigated sociodemographic, family, and behavioral factors associated with earlier exposure in preschool-aged children. We hypothesized that the timing of exposure would vary according to device-specific family and parental factors.

**Methods:**

Cross-sectional data from 892 Portuguese children aged 2 to 6 years were analyzed. Parents reported their children’s age at first exposure to television, video game consoles, computers, tablets, and smartphones. Children not yet exposed were treated as censored observations. Kaplan-Meier analyses estimated the median age at first exposure, and Cox proportional hazards models examined associations with child sex, parental education, parental screen time, and the presence of older siblings.

**Results:**

Among 892 children, exposure to digital devices was nearly universal (n=889, 99.7%), with television being the most common device. The median age at first exposure to any device was 12 months, with 581 (65.1%) children exposed by this age. Timing of exposure varied substantially across devices, with the earliest exposure observed for television, followed by smartphones and tablets, whereas computers and video game consoles were introduced later. Associations with earlier exposure differed by device. Earlier video game exposure was associated with being male and having older siblings. Parental screen time was statistically associated with earlier exposure to computers and tablets, although the magnitude of these associations was small. Smartphone exposure was associated with higher paternal education. No consistent factors were observed for television or overall device exposure.

**Conclusions:**

Digital device exposure begins during infancy and is widespread by the preschool years, but the timing and associated factors differ across devices. These findings suggest that early digital exposure is not a uniform behavior and support the importance of considering device-specific patterns when examining children’s early media experiences.

## Introduction

Access to and use of digital devices have increased markedly in recent decades [1]. Children are engaging with digital devices at increasingly younger ages [1-3], often beginning in infancy through video communication with family members [4]. In addition to direct use, young children are frequently exposed indirectly through background television [5] or when caregivers use digital media to calm or occupy them [6]. As a result, children aged 5 years and younger have become one of the fastest-growing groups of digital media users [7-9]. In Portugal, average screen time increased substantially between 2009/2010 and 2016/2017, reaching 142 minutes per day in preschool-aged children and 173 minutes per day in primary school-aged children [10]. Exceeding recommended screen time is more common among boys, although differences vary by device [10,11].

Importantly, exposure can begin well before the preschool years. Previous research has documented digital media use among children aged under 2 years, with children aged 8 to 36 months already showing substantial exposure to digital devices [8,12]. Similarly, among children aged 3 to 6 years, younger age has been associated with earlier introduction to digital devices [13]. These findings reinforce that digital exposure is established early in childhood and highlight the importance of understanding when children are first introduced to different types of devices.

Early childhood is a critical period for cognitive, behavioral, and socioemotional development, with long-term implications for health and life outcomes [14]. Excessive screen exposure during this period has been associated with poorer attention [15], emotional, behavioral, and sleep-related difficulties [16-18], lower language skills and academic performance [12,19], and adverse physical and mental health outcomes [20]. Beyond total screen time, the timing of first exposure may also be important. Early screen exposure and excessive screen time are independently and interactively associated with lower health-related quality of life and higher risks of behavioral problems [21]. Children introduced to digital devices later also appear less likely to engage in frequent use during adolescence [22]. At the same time, the effects of digital media exposure may depend not only on duration but also on content and context, with some evidence suggesting that educational media may support learning and development under appropriate conditions [23-26].

These concerns have informed recommendations to limit digital media exposure during the first years of life. The World Health Organization (WHO) recommends no sedentary screen time for infants under 1 year and for 1-year-old children, and no more than 1 hour per day for children aged 2 to 4 years, with less being preferable [27]. A recent systematic review of 41 recommendations from international organizations, governments, and professional societies found substantial convergence regarding screen exposure in early childhood, with 20 of 22 recommendations addressing children younger than 2 years recommending zero screen exposure, and most recommendations for children aged 2 to 5 years recommending a maximum of 1 hour per day [28]. This review also highlights that recommendations increasingly address not only the quantity of screen exposure but also its quality and context, including age-appropriate content, parental engagement, and avoidance of digital media as background exposure. Recent European pediatric guidance similarly emphasizes age-appropriate restrictions and consideration of the potential displacement of sleep, physical activity, social interaction, and other developmentally important activities [29].

Despite increasing attention to early digital exposure, important gaps remain. Most studies focus on total screen time rather than age at first exposure, although earlier initiation may shape later patterns of use [22,30]. Studies also rarely distinguish between different types of devices, despite differences in accessibility, interactivity, and developmental relevance (eg, passive television viewing versus interactive mobile devices) [31,32]. Furthermore, although some correlates of screen time have been identified, including among young children [33], there is limited evidence on the factors associated specifically with earlier initiation of digital device use. In particular, relatively little is known about whether the timing of first exposure differs across devices and how it relates to sociodemographic characteristics, family structure, and parental digital behavior. Addressing these gaps may help clarify how children’s digital environments are established within the family and identify factors that could be considered in early prevention and digital well-being strategies.

Therefore, this study aimed to examine the age at first exposure to multiple digital devices (television, computers, video game consoles, tablets, and smartphones) in preschool-aged children using survival analysis methods to account for censored observations. Additionally, associations between sociodemographic (child sex and parental education), family (older siblings), and behavioral (parental screen time) factors and the timing of first exposure to digital devices were assessed. We hypothesized that children would be exposed first to television and that the timing of exposure to different devices would vary according to device-specific parental and family factors.

## Methods

### Study Design and Sampling

This cross-sectional study used baseline data from the ScreenHealth longitudinal project [34]. The project recruited preschool-aged children attending public and private preschools in the central region of Portugal. A total of 132 childcare centers and preschools were initially assessed for eligibility. Of these, 21 were ineligible and 51 declined participation, primarily due to a lack of time, involvement in other research projects, or concerns about participating in a longitudinal study. Consequently, 60 childcare centers and preschools participated in the study. Data were collected between April and June 2024. All children attending the participating institutions were invited to take part, and their parents or legal guardians received written information about the study, an informed consent form, and a paper-based questionnaire to complete at home. Completed questionnaires were returned to the schools in sealed envelopes to ensure confidentiality.

A total of 2491 parents provided written informed consent (parental participation rate: n=1050, 42.2%). Questionnaires were screened for completeness before data entry. Of these, 158 children were excluded due to missing information regarding exposure status for all assessed devices (television, computer, video games, tablet, and smartphone), resulting in a final analytical sample of 892 children. The included participants did not differ from excluded cases with respect to available baseline characteristics.

### Ethical Considerations

It was conducted in accordance with the guidelines of the Declaration of Helsinki and was approved by the Ethics Committee of the Institute for Interdisciplinary Research, University of Coimbra (Reference 1_ID312; July 26, 2023), and supported by the University of Coimbra’s Data Protection Officer. A signed informed consent form was obtained from the parent or legal guardian of each participant.

### Variables and Covariates

For each digital device (television, video game console, computer, tablet, and smartphone), parents were asked whether their child had ever used the device (yes/no). If the response was affirmative, parents reported the child’s age at first exposure in years and/or months. Reporting age in years and/or months, rather than requiring exact dates, was intended to facilitate parental recall. Children who had never been exposed to a given device at the time of data collection were classified as nonexposed, and their age at assessment was treated as a right-censored observation in the survival analyses. A composite variable (“any device”) represented the earliest exposure to any digital device.

Sociodemographic variables included the child’s sex and parental education. Parents reported their education level, categorized as below university vs university (bachelor’s degree or higher). Family context was assessed based on the presence of older siblings (yes/no).

Parental screen time was assessed separately for mothers and fathers across devices on weekdays and weekends. For each parent, reported screen time was converted from categorical responses to estimated minutes of use and combined across devices and days according to the predefined study scoring procedure. The resulting measure represented total parental screen time in minutes per day and was entered as a continuous variable in the models. The original question wording and response formats to assess age at first exposure to each digital device and parental screen behavior are included in Multimedia Appendix 1.

The questionnaire also recorded the respondent’s identity. Among the 892 questionnaires, most were completed by mothers (n=668, 75.3%) or fathers (n=61, 6.9%), while 153 (17.2%) were completed jointly, including by both parents (n=151, 17.0%), the mother and stepfather (n=1, 0.1%) or the father and stepmother (n=1, 0.1%). A small proportion were completed by a grandmother (n=2, 0.2%) or a sibling (n=3, 0.3%).

### Statistical Analysis

Child age was modeled in months. Descriptive statistics included means and SD for continuous variables and frequencies (%) for categorical variables, with analyses also stratified by sex. Kaplan-Meier survival analyses estimated age at first exposure to each device, accounting for censored observations. Differences between boys and girls were assessed using the log-rank (Mantel-Cox) test. Median age at first exposure and 95% CIs were obtained (Kaplan-Meier estimates) when at least 50% of children had experienced the event. For devices with lower exposure prevalence (<50%), median age among exposed children and IQR were reported. Exposure prevalence differences were tested using chi-square tests. Cox proportional hazards regression examined associations of sociodemographic, family, and behavioral factors with the timing of first exposure. Models were estimated using complete-case analysis; therefore, participants with missing data on any model covariate were excluded from the corresponding regression analysis. Survival analysis was selected because not all children had experienced the event (first exposure) at the time of assessment. Standard linear regression approaches would require excluding these children or treating their age at first exposure as observed, potentially introducing bias. Survival methods allow inclusion of all participants by appropriately handling children who had not yet experienced the event as right-censored observations while estimating the timing of first exposure. The event of interest was first exposure to each digital device. Time-to-event was defined as age in months. Children who had not yet experienced the event at the time of assessment were treated as right-censored observations. To facilitate interpretation of the associations between parental screen time and age at first exposure, parental screen time was rescaled from minutes per day to 60-minute units before inclusion in the Cox proportional hazards regression models. Thus, hazard ratios (HRs) for parental screen time represent the relative change in the hazard of first exposure associated with each additional 60 minutes per day of parental screen time. Model 1 included child sex, parental education, and older siblings; model 2 additionally included parental screen time. Multicollinearity was assessed using variance inflation factors, which ranged from 1.21 to 1.28. Analyses were conducted in SPSS software (version 28.0; IBM Corp) with statistical significance set at *P*<.05.

## Results

The sample included 892 children (n=477, 53.5% boys) with a mean age of 55.9 (SD 12.3) months (Table 1). Most mothers (552/871, 63.4%) and approximately half of fathers (399/829, 48.1%) had a university-level education. Overall, 373 (41.8%) children had at least one older sibling. Mean parental screen time was 267.0 (SD 125.5) minutes per day for mothers and 318.7 (SD 153.8) minutes per day for fathers. Descriptive characteristics were broadly similar between boys and girls.

**Table 1.** Sample characteristics (N=892).

Variable	Total	Male	Female
Child characteristics			
Sex, n/N (%)			
Male	477/892 (53.5)	—^a^	—
Female	415/892 (46.5)	—	—
Age (months), mean (SD)	55.9 (12.3)	56.1 (12.1)	56.7 (12.6)
Family sociodemographic, n/N (%)			
Mother’s education			
Below university	319/871 (36.6)	173/463 (37.4)	146/408 (35.8)
University	552/871 (63.4)	290/463 (62.6)	262/408 (64.2)
Father’s education			
Below university	430/829 (51.9)	219/444 (49.3)	211/385 (54.8)
University	399/829 (48.1)	225/444 (50.7)	174/385 (45.2)
Family context, n/N (%)			
Older siblings (yes)	373/892 (41.8)	193/477 (40.5)	180/415 (43.4)
Parental screen behavior (min/d), mean (SD)			
Mother’s screen time	267.0 (125.5)	261.2 (117.4)	273.5 (133.9)
Father’s screen time	318.7 (153.8)	311.6 (145.0)	326.6 (162.8)

a Not available.

Exposure to digital devices was highly prevalent among 892 children (Table 2). Almost all children had been exposed to at least one device (n=889, 99.7%) and to television (n=887, 99.4%). Smartphone (n=742, 83.2%) and tablet (n=513, 57.5%) use was also common, whereas exposure to computers (n=287, 32.2%) and video games (n=179, 20.1%) was less frequent. The only significant sex difference in the prevalence of exposure was observed for video games, which were more common among boys than girls (25.4% vs 14.0%, respectively; *χ*²_1_=17.95, *P*<.001).

**Table 2.** Age at first exposure to digital devices and differences by child sex.

Device	Exposed, n/N (%)	Age (months)		Log-rank *P* value
		Median (IQR)^a^	Median (95% CI)^b^	
Television				.97
Total	887/892 (99.4)	12.0 (12.0-18.0)	12.0 (11.9-12.1)	
Male	474/477 (99.4)	12.0 (12.0-18.0)	12.0 (11.9‐12.1)	
Female	413/415 (99.5)	12.0 (10.5-19.5)	12.0 (11.9‐12.1)	
Video game				N/A^c^
Total	179/892 (20.1)	48.0 (36.0-60.0)	—^d^	
Male	121/477 (25.4)	48.0 (36.0-60.0)	—	
Female	58/415 (14.0)	48.0 (36.0-60.0)	—	
Computer				N/A
Total	287/892 (32.2)	36.0 (24.0-48.0)	—	
Male	155/477 (32.5)	36.0 (24.0-48.0)	—	
Female	132/415 (31.8)	36.0 (24.0-48.0)	—	
Tablet				.69
Total	513/892 (57.5)	36.0 (24.0-36.0)	48.0 (46.4‐49.6)	
Male	276/477 (57.9)	36.0 (24.0-36.0)	48.0 (45.7‐50.3)	
Female	237/415 (57.1)	36.0 (24.0-36.0)	46.0 (43.6‐48.4)	
Smartphone				.86
Total	742/892 (83.2)	24.0 (24.0-36.0)	36.0 (35.1‐36.9)	
Male	401/477 (84.1)	24.0 (24.0-36.0)	36.0 (34.8‐37.2)	
Female	341/415 (82.2)	24.0 (24.0-36.0)	30.0 (27.8‐32.2)	
Any device				.60
Total	889/892 (99.7)	12.0 (12.0-18.0)	12.0 (11.9‐12.1)	
Male	474/477 (99.4)	12.0 (12.0-18.0)	12.0 (11.9‐12.1)	
Female	415/415 (100)	12.0 (12.0-24.0)	12.0 (11.9‐12.1)	

a Descriptive analyses done among the exposed only.

b Kaplan-Meier.

c Not applicable, since less than 50% of the sample had experienced the event.

d Not available.

Age at first exposure was also analyzed in Table 2. Kaplan-Meier analyses showed that exposure to television and to any digital device occurred very early, with a median age of 12 months. Median age at first exposure was 36 months for smartphones and 48 months for tablets. No significant differences between boys and girls were observed in the timing of exposure for any device (all log-rank *P*>.05). For computers and video games, fewer than 50% of children had experienced the event, and therefore the Kaplan-Meier median age at first exposure could not be estimated. Among exposed children, the descriptive median age at first exposure was 36 months for computers and 48 months for video games.

Kaplan-Meier curves illustrating age at first exposure to digital devices by sex are presented in Figure 1. The curves show a steep decline during the first years of life for television and overall device exposure, indicating that most children were exposed at very early ages. In contrast, more gradual declines were observed for smartphones and tablets, reflecting later exposure. Across all devices, the curves for boys and girls largely overlapped, indicating the absence of significant sex differences in the timing of exposure.

**Figure 1.**
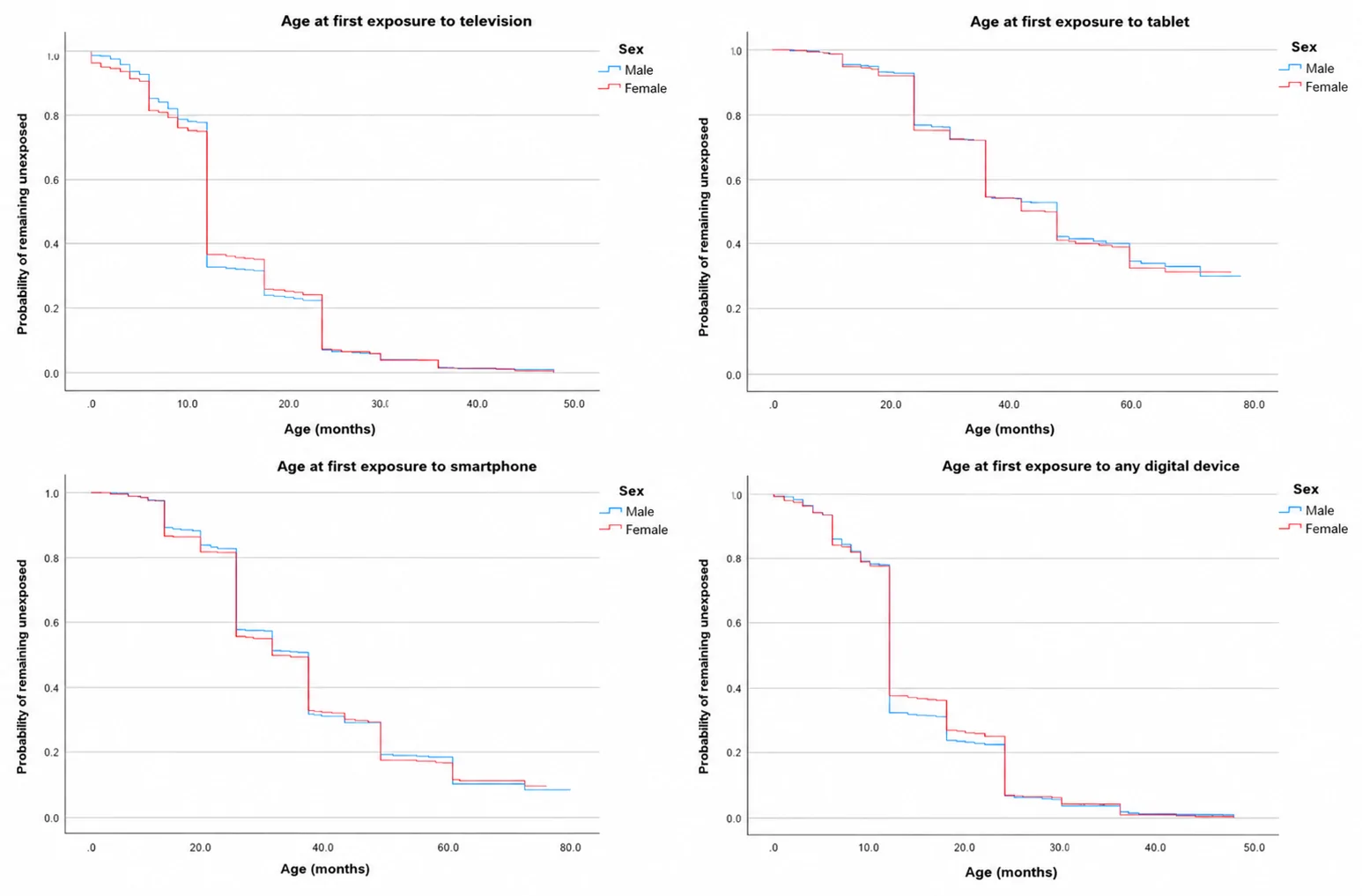
Kaplan-Meier curves for age at first exposure to digital devices, by child sex. The curves represent the probability of remaining unexposed to each device over time (in months). Differences between boys and girls were assessed using log-rank (Mantel-Cox) tests with no significant differences observed for any outcome.

Results from Cox regression analyses showed that associations varied according to the type of device, with generally small to moderate effect sizes. In model 1 (data not shown), which included child sex, parental education, and older siblings, higher paternal education was associated with a modestly earlier television exposure (HR 1.19, 95% CI 1.02‐1.38), corresponding to approximately a 19% higher likelihood of earlier exposure. For video game exposure, boys had a higher hazard of first exposure than girls (HR 1.87, 95% CI 1.36‐2.57), and children with older siblings also had a moderately higher hazard of first exposure (HR 1.53, 95% CI 1.13‐2.07). For smartphone use, lower maternal education was associated with a lower hazard of first exposure (HR 0.82, 95% CI 0.69‐0.97), whereas higher paternal education was associated with a moderately higher hazard (HR 1.29, 95% CI 1.09‐1.52). No statistically significant associations were observed for computer, tablet, or overall device exposure in this model.

When additionally adjusting for parental screen time (model 2–fully adjusted), most associations remained small in magnitude (Table 3). For video game exposure, boys remained approximately twice as likely to be exposed earlier than girls (HR 2.02, 95% CI 1.45‐2.80), while having older siblings was associated with a 46% higher hazard of earlier exposure (HR 1.46, 95% CI 1.08‐1.99). Maternal screen time was also associated with earlier video game exposure (HR 1.15, 95% CI 1.06‐1.24), corresponding to a 14.7% higher hazard of earlier exposure for each additional 60 minutes per day of maternal screen time. Maternal screen time was also statistically associated with earlier computer exposure (HR 1.08, 95% CI 1.01‐1.16), although the magnitude of the association was modest. For tablet exposure, higher paternal screen time was associated with earlier exposure (HR 1.10, 95% CI 1.06‐1.15), corresponding to a 10.2% higher hazard per additional 60 minutes per day. For smartphone exposure, higher paternal education remained associated with earlier exposure (HR 1.28, 95% CI 1.07‐1.52), representing a modest association. No statistically significant associations were observed for television or for overall exposure to any device. Results for computer and video game exposure should be interpreted with caution because of the lower proportion of children exposed, which may reduce the precision and stability of the corresponding estimates.

**Table 3.** Factors associated with age at first exposure to digital devices (Cox proportional hazards models)^a^.

Variable	HR^b^ (95% CI)	*P* value
Television		
Sex (reference: female)	1.007 (0.869‐1.167)	.93
Mother education (reference: university)	1.004 (0.850‐1.185)	.97
Father education (reference: university)	1.154 (0.984‐1.355)	.08
Older siblings (reference: number)	1.114 (0.961‐1.291)	.15
Mother screen time	1.008 (0.969‐1.048)	.70
Father screen time	1.021 (0.988‐1.055)	.22
Video game		
Sex (reference: female)	2.018 (1.454‐2.801)	<.001
Mother education (reference: university)	1.158 (0.824‐1.629)	.40
Father education (reference: university)	0.978 (0.704‐1.359)	.89
Older siblings (reference: number)	1.461 (1.075‐1.985)	.02
Mother screen time	1.147 (1.058‐1.244)	.001
Father screen time	1.019 (0.949‐1.094)	.60
Computer		
Sex (reference: female)	1.009 (0.783‐1.299)	.95
Mother education (reference: university)	0.885 (0.659‐1.187)	.41
Father education (reference: university)	0.954 (0.725‐1.254)	.73
Older siblings (reference: number)	0.905 (0.700‐1.169)	.44
Mother screen time	1.082 (1.014‐1.155)	.02
Father screen time	0.982 (0.926‐1.040)	.53
Tablet		
Sex (reference: female)	0.988 (0.815‐1.198)	.90
Mother education (reference: university)	1.110 (0.895‐1.377)	.34
Father education (reference: university)	1.159 (0.941‐1.428)	.17
Older siblings (reference: number)	1.079 (0.890‐1.309)	.44
Mother screen time	1.031 (0.978‐1.086)	.23
Father screen time	1.102 (1.056‐1.149)	<.001
Smartphone		
Sex (reference: female)	0.943 (0.803‐1.107)	.47
Mother education (reference: university)	0.861 (0.716‐1.035)	.11
Father education (reference: university)	1.277 (1.071‐1.523)	.006
Older siblings (reference: number)	0.884 (0.752‐1.038)	.13
Mother screen time	1.016 (0.972‐1.063)	.48
Father screen time	1.034 (0.996‐1.074)	.08
Any device		
Sex (reference: female)	1.044 (0.900‐1.210)	.57
Mother education (reference: university)	1.031 (0.873‐1.218)	.72
Father education (reference: university)	1.124 (0.958‐1.319)	.15
Older siblings (reference: number)	1.073 (0.926‐1.245)	.35
Mother screen time	1.008 (0.969‐1.049)	.69
Father screen time	1.023 (0.990‐1.057)	.18

a Covariates were included simultaneously in the model. Parental screen time was entered as a continuous variable expressed in 60-minute units; therefore, HRs represent the association with each additional 60 min/d of parental screen time.

b HR: hazard ratios.

## Discussion

### Principal Findings

This cross-sectional study examined the age at first exposure to multiple digital devices in preschool-aged children and the association of sociodemographic, family, and behavioral factors with the timing of first exposure. Three main findings emerged. First, exposure to digital devices was nearly universal, particularly for television, followed by smartphones and tablets, with minimal sex differences. Second, exposure occurred very early, with nearly two-thirds of children exposed to at least one digital device by 12 months and more than 90% by 2 years of age. Third, factors associated with earlier exposure varied according to device type, suggesting that early digital exposure is not a uniform developmental event but rather comprises distinct device-specific patterns.

Nearly all children in the sample had been exposed to digital media, with television being the most common form of exposure. Similar patterns have been reported among children aged 8 years and younger in the United States [8] and among children aged 3 to 10 years in Portugal [35]. The increasing use of smartphones and tablets has also been documented in children aged 15 to 36 months [6]. Importantly, evidence from even younger children indicates that digital device use is already established during infancy and the toddler period [12]. The increasing presence of digital media during early childhood is particularly relevant because screen-based activities may displace sleep, physical activity, parent-child interaction, and other experiences important for development [15-21,36]. The only significant sex difference in exposure prevalence in the present study was observed for video games, which were more common among boys, consistent with previous findings [8].

The median age at first exposure to any device was 12 months, largely driven by television. More than half of 892 children (n=581, 65.1%) had been exposed by 12 months and nearly all (829, 92.9%) by age 2. This pattern indicates that digital exposure is commonly initiated well before the preschool period and is broadly consistent with evidence showing that screen exposure can begin during infancy [1,3,12,37,38]. It also contrasts with recommendations that emphasize minimizing or avoiding sedentary screen exposure during the first years of life. The WHO recommends no sedentary screen time for infants younger than 1 year and for 1-year-old children and no more than 1 hour per day for children aged 2 to 4 years, with less being preferable [27]. A recent systematic review of recommendations further demonstrated that guidance generally supports avoiding or minimizing screen exposure in children younger than 2 years, although recommendations vary in their wording and in the extent to which they address content, context, and co-use [28]. Thus, while the present study was not designed to evaluate adherence to specific guidelines, the very early age at first exposure observed in this sample highlights how digital media are introduced during a period in which recommendations generally encourage little or no screen exposure.

The particularly early introduction of television may partly reflect the characteristics of the device and the household environment. Television is commonly a shared household device, often positioned in a central location where it can be seen and heard by multiple family members. Consequently, young children may encounter television content even when they are not intentionally watching it. In contrast, smartphones, tablets, computers, and video game consoles are generally more personal or require more intentional interaction, potentially contributing to their later reported introduction. Previous research has similarly identified television as an important source of early screen exposure [5,8,12]. However, an important distinction should be made when interpreting the present findings: the questionnaire asked parents about the age at which the child first “used” each device but did not distinguish between intentional/direct use and incidental or background exposure. Therefore, the reported age of first television exposure may include both active viewing and indirect exposure to television in the household. This distinction may be particularly important when interpreting the very early median age observed for television and represents an important limitation of the present study.

Later ages at first exposure were observed for computers and video game consoles. This may reflect their more specific functional use and, potentially, greater demands for intentional interaction compared with television. Previous studies suggest that engagement with video games typically begins around the age of 3 years [39,40], while computer use may increase as children approach school entry [41]. Nevertheless, these differences should not necessarily be interpreted as evidence that some devices are inherently more or less appropriate for young children. Rather, they may reflect differences in accessibility, household ownership, intended purpose, and the circumstances in which different devices are used.

A key finding was that factors associated with earlier exposure differed across devices. This pattern suggests that early digital exposure may be better understood as a collection of device-specific practices shaped by different combinations of child, family, and environmental characteristics, rather than as a single developmental event. The accessibility and portability of devices, their role within family routines, and whether they are commonly shared or individually used may influence when children first encounter them. In addition, early digital experiences may be shaped by parental mediation, caregiver practices, sibling influence, and the purposes for which devices are introduced, including entertainment, education, communication, or calming or occupying the child [42-47]. Because these contextual dimensions were not directly assessed in the present study, these mechanisms remain hypotheses rather than explanations demonstrated by our data.

For video games, being male was strongly associated with earlier exposure, whereas having older siblings showed a moderate association. The association with sex is consistent with previous findings [40] and may reflect differences in gaming preferences or opportunities for engagement with video games during childhood. The association with older siblings may indicate that children are introduced to gaming through shared family activities or through access to devices already used by older children. Siblings may therefore represent an important pathway through which digital practices are introduced into the child’s environment, particularly for devices that are less likely to be used by very young children independently. However, because the present study did not assess whether devices were shared with siblings or how exposure occurred, this interpretation remains speculative.

Parental screen time was associated with earlier exposure to video games, computers, and tablets after adjustment, but the magnitude of these associations differed substantially. The associations for maternal screen time with video game and computer exposure and paternal screen time with tablet exposure were statistically significant when screen time was expressed in 60-minute units, but the corresponding HRs indicate relatively modest changes in the likelihood of earlier exposure for each additional hour of parental screen time. These findings should therefore not be interpreted as evidence that parental screen time is a major determinant of children’s early exposure. Instead, parental screen time may reflect a broader family media environment in which children have greater opportunities to observe, access, or participate in digital activities.

Several mechanisms may plausibly contribute to this association. Parents may act as role models, with children becoming familiar with devices through observing their parents’ digital behavior. Parental media use may also be embedded in shared family routines, increasing children’s opportunities to interact with particular devices. Conversely, frequent parental device use may reduce opportunities for direct parent-child interaction or encourage the use of digital devices as a practical means of occupying or calming children [43,47,48]. However, these mechanisms were not directly assessed in this study. In particular, we did not measure parental mediation, co-use, reasons for introducing a device, or whether the child’s exposure occurred while a parent was using the same device. Therefore, parental screen time should be interpreted primarily as an indicator of the broader family digital environment rather than as evidence of a direct causal pathway.

The observed associations for maternal and paternal screen time should also not be interpreted as evidence for distinct maternal or paternal causal effects. Differences between maternal and paternal associations [49] may reflect differences in caregiving roles, time spent with the child, occupational routines, device ownership, or shared variance between parental behaviors. Residual confounding may also contribute. Future studies should therefore examine parental digital behavior together with caregiving time, parental mediation, co-use, and the circumstances under which children are introduced to specific devices.

Smartphone exposure showed a different pattern, remaining associated with paternal education after adjustment for parental screen time. The magnitude of this association was modest, suggesting that paternal educational attainment was associated with, but did not appear to be, a dominant explanation for earlier smartphone exposure. This finding should also be interpreted cautiously because educational attainment is not a direct proxy for socioeconomic status. Educational level may correlate with several interrelated characteristics, including occupational circumstances, parental awareness and beliefs about digital media, access to technology, digital practices, and broader family contexts [50]. The direction of the association also contrasts with some previous studies linking lower parental education to earlier or greater media exposure [44,45,51], although findings across studies are inconsistent [46]. Maternal education was associated with earlier smartphone exposure in the minimally adjusted models but not after adjustment for parental screen time, suggesting that the initial association may partly overlap with other family behavioral characteristics.

Overall, the findings indicate that statistical significance should be considered alongside the magnitude and precision of the observed associations. Some factors, particularly child sex and older siblings for video game exposure, showed comparatively larger associations, whereas parental screen time generally showed smaller associations. This distinction is important because statistically significant associations can arise even when the absolute magnitude of the association is limited, particularly in relatively large samples. Accordingly, the present findings should be interpreted as identifying correlates of earlier exposure rather than establishing major determinants of causal effects. The absence of consistent associations for television and for exposure to any device further suggests that aggregate measures may conceal meaningful device-specific patterns.

An additional consideration is that the present study focused on when children were first exposed to devices, rather than on how those devices were used. We did not distinguish between active and passive use, educational and entertainment content, coviewing with caregivers, or parental mediation. These distinctions are increasingly recognized as important when evaluating the implications of children’s digital media exposure [27,28]. For example, the developmental implications of a child watching television alone may differ from those of a caregiver and child engaging together with age-appropriate content, even if the nominal duration of exposure is similar. Similarly, first exposure to a smartphone may involve video communication, educational content, entertainment, or simply observing a parent’s use of the device. Consequently, the timing of first exposure should not be interpreted as a direct indicator of the quality or developmental impact of that exposure. Rather, it provides information about when digital media become part of children’s environments, which can subsequently be characterized in greater detail in terms of content and context.

### Strengths and Limitations

This study has several strengths. First, it examined the age at first exposure across multiple digital devices, providing a more nuanced characterization of early media exposure than studies that focus solely on total screen time. Second, the use of survival analysis allowed children who had not yet been exposed to a given device at the time of assessment to be appropriately retained as censored observations. This is particularly relevant when studying age at first exposure because excluding unexposed children could systematically bias estimates toward earlier ages. Third, multiple sociodemographic, family, and behavioral characteristics were considered simultaneously, allowing device-specific patterns to be examined.

Several limitations should be acknowledged. First, the cross-sectional design and retrospective assessment of age at first exposure preclude causal inference and may introduce recall bias. Although parents reported the age of first exposure in years and/or months, recalling events that occurred several years earlier may be difficult, particularly for exposures during infancy. Recall error may therefore have affected the precision of estimated ages at first exposure and associations with family characteristics. Because television exposure was particularly early, this issue may be especially relevant to the estimates for television and overall device exposure. Future longitudinal studies beginning during infancy, with prospective recording of first exposure, would reduce reliance on retrospective recall. Second, the questionnaire did not distinguish between active and background exposure. This limitation is particularly relevant for television, which is frequently present in shared household spaces and may be encountered incidentally even when a child is not intentionally viewing it. Thus, the very early age reported for first television exposure may partly reflect background exposure rather than deliberate screen use. Similarly, the study did not collect information on the content viewed, whether exposure was educational or entertainment-oriented, whether parents or siblings coviewed with the child, or the extent of parental mediation. These contextual characteristics may be important for understanding the meaning and potential implications of first exposure.

Third, although respondents were asked who completed the questionnaire, the study did not specifically assess whether children were regularly cared for by other caregivers, such as grandparents or nannies, or whether parental separation or other family arrangements could have affected the respondent’s knowledge of the child’s first exposure. Most questionnaires were completed by mothers, fathers, or both parents together, while only a very small proportion were completed by other family members. Nevertheless, residual reporting error cannot be excluded when the respondent was not continuously responsible for the child’s care. Fourth, selection bias cannot be excluded. Participation occurred through educational institutions and required parental participation, and participating families may differ from nonparticipating families in characteristics related to digital media practices, such as educational attainment, access to digital technologies, or interest in child health and research. Such differences may limit the generalizability of the findings to the wider population of preschool-aged children.

Fifth, the proportion of missing data was modest for most variables; however, parental screen time had a higher proportion of missing data, resulting in a reduced sample size for the fully adjusted Cox regression models due to the use of complete-case analysis. This reduction in sample size may have decreased statistical power and could have introduced bias if missingness was related to both parental screen behavior and children’s digital exposure. Finally, the study examined the timing of first exposure rather than subsequent patterns of use or developmental consequences; therefore, the findings cannot establish whether earlier exposure to a particular device leads to poorer developmental, behavioral, or health outcomes, nor can they determine whether the observed associations reflect causal family processes or broader characteristics of the household environment. Longitudinal studies incorporating prospective measures of exposure, device-specific content and context, co-use, parental mediation, and developmental outcomes are needed to clarify these pathways.

### Conclusions

Exposure to digital devices occurred at very early ages, and it was nearly universal by the preschool years. Importantly, the timing of first exposure differed substantially across devices, with television being introduced particularly early, while smartphones, tablets, computers, and video game consoles followed different trajectories. The factors associated with earlier exposure also varied by device, indicating that early digital exposure should not be considered a single, uniform developmental event.

These findings provide a more differentiated perspective on early digital media exposure by showing that the timing of children’s first encounter with a device may depend on the type of technology and the family environment in which it is introduced. At the same time, the observed associations should not be interpreted as causal determinants, particularly where effect sizes were small. From a family and public health perspective, these findings support approaches that consider the broader home digital environment—including parental and sibling practices, opportunities for co-use and mediation, and the circumstances in which different devices are introduced—rather than focusing exclusively on total screen time or on individual devices. Future longitudinal research should build on this approach by examining how the timing of first exposure interacts with content, context, coviewing, and subsequent patterns of use to influence children’s development and well-being.

## Supplementary material

10.2196/100072 Multimedia Appendix 1 Questionnaire items used to assess age at first digital-device exposure and parental screen time.

## Abbreviations

HR: hazard ratio
WHO: World Health Organization
