# Supplementary material for: Determinants of Age at First Exposure to Digital Devices Among Children Aged 2 to 6 Years: Survival Analysis
Source: J Med Internet Res. 2026 Oct 28;28:e100072. doi: 10.2196/100072 (PMC13716357; doi:10.2196/100072)
Supplement: Supplementary file 1 [file jmir-v28-e100072-s001.docx]

**Supplementary Material 1 – Questionnaire items used to assess age at first digital-device exposure and parental screen time**

**Note:** The questionnaire was originally administered in Portuguese. The items below are provided in English for transparency and reproducibility and represent an English translation of the original questionnaire items.

***Section A – Child’s age at first exposure***

**At what age did your child first use a digital device, such as:**

**Television:** ______ year(s) and ______ month(s)	☐ Never watched

**Computer:** ______ year(s) and ______ month(s)	☐ Never used

**Videogame console:** ______ year(s) and ______ month(s). ☐ Never used

**Tablet:** ______ year(s) and ______ month(s). ☐ Never used

**Smartphone:** ______ year(s) and ______ month(s)	☐ Never used

***Section B – Parental screen time***

**Thinking about a typical week, how much time do you spend using electronic devices such as:**

The following question was administered **separately for the mother and the father**. The same response options were provided for each device and each day type.

**Television**

**Weekday (Monday–Friday)**
☐ None
☐ Up to 1 hour
☐ 1 to 2 hours
☐ 2 to 3 hours
☐ 3 to 4 hours
☐ More than 4 hours

**Saturday**
☐ None
☐ Up to 1 hour
☐ 1 to 2 hours
☐ 2 to 3 hours
☐ 3 to 4 hours
☐ More than 4 hours

**Sunday/Holiday**
☐ None
☐ Up to 1 hour
☐ 1 to 2 hours
☐ 2 to 3 hours
☐ 3 to 4 hours
☐ More than 4 hours

**Videogame console**

**Weekday (Monday–Friday)**
☐ None
☐ Up to 1 hour
☐ 1 to 2 hours
☐ 2 to 3 hours
☐ 3 to 4 hours
☐ More than 4 hours

**Saturday**
☐ None
☐ Up to 1 hour
☐ 1 to 2 hours
☐ 2 to 3 hours
☐ 3 to 4 hours
☐ More than 4 hours

**Sunday/Holiday**
☐ None
☐ Up to 1 hour
☐ 1 to 2 hours
☐ 2 to 3 hours
☐ 3 to 4 hours
☐ More than 4 hours

**Computer (desktop and/or laptop)**

**Weekday (Monday–Friday)**
☐ None
☐ Up to 1 hour
☐ 1 to 2 hours
☐ 2 to 3 hours
☐ 3 to 4 hours
☐ More than 4 hours

**Saturday**
☐ None
☐ Up to 1 hour
☐ 1 to 2 hours
☐ 2 to 3 hours
☐ 3 to 4 hours
☐ More than 4 hours

**Sunday/Holiday**
☐ None
☐ Up to 1 hour
☐ 1 to 2 hours
☐ 2 to 3 hours
☐ 3 to 4 hours
☐ More than 4 hours

**Tablet**

**Weekday (Monday–Friday)**
☐ None
☐ Up to 1 hour
☐ 1 to 2 hours
☐ 2 to 3 hours
☐ 3 to 4 hours
☐ More than 4 hours

**Saturday**
☐ None
☐ Up to 1 hour
☐ 1 to 2 hours
☐ 2 to 3 hours
☐ 3 to 4 hours
☐ More than 4 hours

**Sunday/Holiday**
☐ None
☐ Up to 1 hour
☐ 1 to 2 hours
☐ 2 to 3 hours
☐ 3 to 4 hours
☐ More than 4 hours

**Smartphone**

**Weekday (Monday–Friday)**
☐ None
☐ Up to 1 hour
☐ 1 to 2 hours
☐ 2 to 3 hours
☐ 3 to 4 hours
☐ More than 4 hours

**Saturday**
☐ None
☐ Up to 1 hour
☐ 1 to 2 hours
☐ 2 to 3 hours
☐ 3 to 4 hours
☐ More than 4 hours

**Sunday/Holiday**
☐ None
☐ Up to 1 hour
☐ 1 to 2 hours
☐ 2 to 3 hours
☐ 3 to 4 hours
☐ More than 4 hours

**Note:** The parental screen-time questions above were completed separately for the **mother** and **father**. Parental screen time was subsequently derived from the reported device-specific and day-specific categories according to the study's predefined scoring procedure.

**Please indicate who completed the questionnaire:**

☐ Mother
☐ Father
☐ Grandmother
☐ Grandfather
☐ Brother/Sister
☐ Other. Please specify the relationship to the child: ______________________
